# Savoring every drop – Vampire or Mosquito?

**DOI:** 10.1186/cc13884

**Published:** 2014-05-21

**Authors:** Dania Patricia Fischer, Kai D Zacharowski, Patrick Meybohm

**Affiliations:** 1 Department of Anaesthesiology, Intensive Care Medicine and Pain Therapy, University Hospital Frankfurt, Theodor-Stern-Kai 7, 60590 Frankfurt am Main, Germany

## Abstract

Blood safety with respect to infectious complications has reached very high standards. Nevertheless, reports on transfusion-associated morbidity and mortality gain momentum. Multidisciplinary patient blood management programs can minimize unnecessary exposure to allogeneic blood products by strengthening and conserving patients’ own resources. This article outlines concepts designed to maintain hemoglobin concentration, to optimize hemostasis, and to minimize blood loss in ICUs. These measures prevent or at least alleviate hospital-acquired anemia, reduce the need for blood transfusions, and therefore have great potential to improve patient safety and medical outcome.

## Introduction

Blood is the most precious and eclectic of fluids. Blood transports oxygen and nutrients throughout the body, heals our wounds, connects our organs, and protects us from dangerous pathogens; it is the essence through which we can remain healthy and able. Not surprisingly, the shortage of blood and especially of the associated hemoglobin - manifested clinically as anemia - negatively affects patient outcomes [1-5]. Anemia is a very common phenomenon, particularly among critically ill patients. Approximately two thirds of the patients already show a hemoglobin concentration of below 120 g/L on admission to the ICU, and after 1 week 97% are anemic [3,5,6]. This condition often results from a combination of nutritional deficiency, hemolysis, myelosuppression, renal insufficiency, and comorbidities. Unfortunately, there are no immediate remedies for anemia without side effects. Blood safety with respect to infectious complications might have reached very high standards [7], but reports on transfusion-associated morbidity and mortality continue to abound. Evidence increasingly suggests that the transfusion of even a single allogeneic red blood cell (RBC) unit can be associated with increased morbidity and mortality because of infectious, immunological, pulmonary, and thromboembolic complications [8-11]. The creation of a suitable synthetic blood mimetic has long been sought after, yet this goal remains elusive.

This leaves practitioners facing a dilemma. On one hand, anemia should be reconciled in order for the body to function at optimal levels, yet the one immediate solution of transfusing allogeneic RBCs may harm the patient further by inducing additional adverse events. This dilemma is arguably most pressing in the ICU because patients are sickest and the close surveillance of the patients requires collection of blood for testing. However, this renders patients even more vulnerable to anemia. Shockingly, weekly blood loss due to phlebotomy and laboratory blood sampling to monitor coagulation, organ function, and acid–base status or to screen for nosocomial infections has been reported to range from 340 to 660 mL in ICU patients [12,13]. Similar observations prompted John F Burnum in 1986 to provocatively refer to physicians as ‘medical vampires’ [14]. Despite our good intentions, we as medical care providers often add considerably to the development of anemia by close laboratory surveillance and other blood-shedding therapeutic measures, such as dialysis.

Particularly in the setting of ICUs, a multicomponent strategy to prevent or at least ameliorate hospital-acquired anemia is needed. There are many angles from which we can tackle this issue while possibly improving outcomes and minimizing additional complications. Areas where a new standard of care could be most beneficial include the improvement of diagnostic and therapeutic procedures and the optimization of coagulation management and hemotherapy. Together, we should seek every possibility to reduce our ‘appetite’ for blood and move from being ‘medical vampires’ to ‘medical mosquitoes’.

## Blood sampling volume and frequency

The Sepsis Occurrence in Acutely Ill Patients study reported a positive correlation between severity of organ dysfunction, the number of blood draws, and the total volume drawn [5]. Admittedly, more iatrogenic blood loss was recorded in the sicker patient groups compared with relatively healthier ones, and this may confound the results as these patients were *per se* more likely to suffer from organ dysfunction due to their underlying condition. Nevertheless, it appears that iatrogenic blood loss has a negative impact on patient outcomes and therefore offers an area with great potential for improvement.

However, there is significant variation between ICUs: demographic and clinical characteristics of patients, varying surgical partners, local standards of care, and laboratory performances are but a few factors that have a large impact on the volume of blood losses. An informative first step is therefore for each hospital to keep a record on the frequency and volume of blood drawings for each patient. A simple visualization is a solid foundation to build upon in order to design methods to effectively reduce blood losses. It can also serve as a baseline to quantify and compare the effect of tailor-made solutions in instances in which a common deficiency is identified at a regional or even national level.

The frequency, volume, and process of blood drawings are rational first steps for consideration in constructing better standards of care. Firstly, scattershot laboratory testing should be avoided by carefully evaluating and streamlining the commonly used practice parameters. Establishing a task force of interdisciplinary local experts representing critical care medicine, surgery, internal medicine, infectious diseases, neurology, laboratory medicine/microbiology, and nursing staff is one approach to this. This task force should establish a consensus on parameters and frequency of laboratory testing in common situations to guide clinical practice. The consensus could be based upon evidence-based recommendations and expert opinion. An example for this is a utilization management intervention, which reduced unnecessary testing in a coronary care unit established by a multidisciplinary team at Massachusetts General Hospital (Boston, MA, USA) [15]. The team viewed the routine determination of ‘extended’ chemistries, such as calcium, magnesium, and phosphorus, as unsubstantiated practice in coronary care patients. The team developed guidelines and computerized order template designs discouraging the routine measurements of these electrolytes and also of arterial blood gases (ABGs) in patients not receiving ventilatory support. Tests for sodium, potassium, chloride, and complete blood counts were recommended to be ordered only once per day. Analysis of ABG measurement was recommended only after significant changes in minute ventilation, fall in oxygen saturation, or significant changes in clinical condition. Wang and colleagues [15] specifically emphasized that blood gases did not have to be assessed with every change in ventilator setting. They were thus able to demonstrate a significant reduction in routine testing without change in clinical outcomes and saw scope for further reduction. However, the study was not powered to assess differences in clinical outcomes, historical controls were used, and the measures of severity of illness were imprecise.

Another example to improve physician test ordering might be a guideline on the evaluation of patients who develop early postoperative fever in the ICU [16]. Fever is a common phenomenon during the initial 48 hours after surgery and usually non-infectious in origin under the precondition that sterility was kept and no aspiration occurred. The guideline by O’Grady and colleagues [16] therefore recommends caution with regard to taking blood cultures in the work-up of early postoperative fever.

Recommendations on the frequency of laboratory testing should also take into account the half-life and appropriateness of clinical biomarkers: C-reactive protein (CRP), for instance, has a half-life of 19 hours and cannot be recommended as an aid to the initiation or discontinuation of any antibiotic in adults, as described by Dupuy and colleagues [17]. CRP values can never be diagnostic on their own and can be interpreted only at the bedside, in full knowledge of all other clinical and pathological results [18]. Besides, laboratory tests should be repeated only if indicated: clinicians should refrain from using prepackaged orderings and move on to hand-picked and also consider the inherent standard deviation of laboratory findings when ordering tests. In this regard, physicians should be mindful that false-positive results need control and therefore should order only the minimum requirement, thus reducing overall burden on the health-care system and reducing the risk of negative outcomes for the patient. We believe that there is great savings potential of restrictive versus standard laboratory testing.

Moreover, the process of blood drawing itself might offer scope for improvement. We encourage the introduction of in-line blood sampling devices, which can also be combined with chemistry monitors. Widness and colleagues [19], for example, could reduce cumulative phlebotomy loss by one fourth in a randomized, controlled, prospective trial on preterm infants: monitor group (n = 46) and control group (n = 47). An in-line bedside monitor was used that withdraws blood through an arterial catheter; analyzes blood gases and sodium, potassium, and hematocrit levels; and returns the sample to the patient. Through the use of in-line blood sampling devices, the same quality of care can be maintained without any increased risk of infection compared with standard arterial and venous lines while less blood is wasted [12,20]. We would argue that in-line blood sampling devices represent a realistic and effective tool in saving patients’ blood.

**Figure 1:**
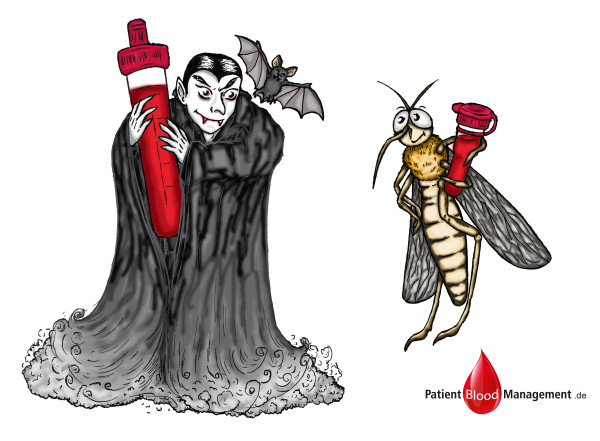
**Appetite for blood: medical vampire or medical mosquito?** Illustration by Pia Ockelmann.

Increased practitioner education on laboratory requirements may also reduce blood wastage. The blood diagnostics unit at the University Hospital Frankfurt, Germany, for instance, requires specimens of only 30 μL for the quantification of procalcitonin; the dead space volume needed to prime the system, however, is 300 μL. Technological innovations in the future may further reduce dead space volume requirements, and the improved preservation of blood specimens for potential subsequent use would be another beneficial area to explore. The sensible use of analytical chemistry techniques and, where applicable, the introduction of pediatric vials (having a capacity of less than 100 μL) should be considered. One study has shown that using pediatric blood collection tubes nearly halved the blood loss associated with diagnostic testing (reduction of 47%) without compromising laboratory test procedures and test quality [21]. Figure 1 illustrates adult (‘vampire’s appetite’) versus such pediatric (‘mosquito’s appetite’) test tubes. In this regard, using smaller tubes may have the potential to reduce the severity of phlebotomy-induced anemia in adults. However, the drawback of this is that smaller test tubes bind more human resources as they usually require manual handling, thereby increasing costs and potentially delaying analysis. Point-of-care (POC) microanalysis such as bedside tests, on the other hand, also often requires less than 500-μL specimens and has the additional advantage of short turnaround times for results, reducing staff time requirements. However, the nearness of the tests might increase the number of tests done.

Additionally, non-invasive tools for hemodynamic monitoring should be used wherever possible as indwelling catheters have been shown to increase blood testing [22]. Transcutaneous measurements of hemoglobin can also be considered, although results from non-invasive analysis might deviate from actual values which render these methods potentially hazardous [23,24]. Further factors such as vasopressor therapy may interfere and represent another serious limitation for non-invasive hemoglobin monitoring in many ICU patients [25].

## Blood-sparing techniques

Continuous dialysis is a common requirement in critical care patients and necessitates sufficient anticoagulation to prevent blood clotting: premature clotting of the dialysis circuit leads to increased blood loss for the patient. On the other hand, anticoagulation itself inherently increases bleeding risk. One therapeutic strategy that extends filter lifetime without increasing the risk of bleeding is the use of citrate-anticoagulation during dialysis. Citrate is administered in the extracorporeal circuit and chelates the calcium required in the coagulation cascade. It extends circuit survival time and filter lifetime [26,27] and furthermore is associated with a significant decrease in bleeding - relative risk (RR) 0.34, 95% CI 0.17 to 0.65 - compared with standard anticoagulation with heparin [28]. However, additional equipment and training of staff are required initially. Once citrate-anticoagulated dialysis is implemented, complication rates are low, making initial investments on monetary and human resources worthwhile in our view.

Cell salvage is another possibility which can be explored. Post-operative blood recovery techniques using cell savers are not applicable in many ICU patients but may be useful in selected cases with the critically ill. In particular, cell salvage should be one of the main cornerstones within the first hours after major surgery in coagulopathic cardiac surgery patients with increased blood loss in order to reduce the need for allogeneic RBC transfusion in ICUs.

## Coagulation management and hemotherapy

Hemostatic abnormalities are common among critically ill patients and often are of multifactorial origin [29]. Treatment decisions are increasingly aided by bedside coagulation monitoring, which provides reliable and fast testing of the overall hemostatic function. Goal-directed algorithms based on results from thrombelastography and rotational thromboelastometry have been shown to reliably regulate coagulopathy in studies on trauma and cardiac patients [30]. However, owing to the nature of diseases encountered in ICUs, interventions that are successful in other settings may not be relevant in the ICU setting. Furthermore, the costs of acquisition and provision of trained staff are high. But although studies on POC-guided hemotherapy algorithms in the treatment of critically ill patients are still pending and initial costs may be a deterrent, the potential to curtail blood losses is high. Coagulation management ideally should be based on a combination of aggregometric and viscoelastic diagnostic methods integrated into an algorithm already validated in other medical settings. The algorithm should emphasize the need to maintain optimal hemostatic conditions with normal pH, temperature, and calcium levels. Furthermore, it should include anti-fibrinolytic agents such as tranexamic acid and conclude with the use of recombinant activated factor VII in selected cases refractory to standard treatment [31,32]. This algorithm should also give an overview of indications for adequate hemotherapy.

RBC transfusion is a common intervention in ICU patients; approximately one third of critically ill patients receive a blood transfusion during their stay in the ICU [33]. However, RBC transfusions have been associated with worse outcomes in several populations of patients, including critically ill patients [3,5,34]. This set off a shift in transfusion policy toward a more restrictive approach encompassing lower transfusion thresholds in the critically ill [35]. Most importantly, it has been recognized that hemotherapy should be individualized in order to carefully balance the risks of allogeneic blood products with the risks of low hemoglobin levels in each single case. Presumably, not only inter-individual but also intra-individual variation exists in the tolerance toward anemia depending on metabolic activity, volume status, physiological reserve, dynamics of the anemia, and other health issues. Generally, normovolemic anemia is better tolerated than hypovolemic anemia and chronic anemia (for instance, due to sepsis) is better tolerated than acute anemia in cases of severe uncontrolled bleeding. Therefore, transfusion decisions should be based on both symptoms as well as laboratory results such as hemoglobin concentration.

Several prospective studies on restrictive versus liberal transfusion strategies in critically ill patients exist. However, there is little evidence on safe transfusion strategies for patients with cardiovascular diseases, causing a lot of insecurity in the care of such patients [36,37]. The TRICC (Transfusion Requirements in Critical Care) trial showed in 838 ICU patients that a restrictive transfusion threshold (hemoglobin (Hb) trigger 70 g/L, target 70 to 90 g/L) compared to a liberal one (Hb trigger 100 g/L, target 100 to 120 g/L) is at least as effective [38]. However, the trial did not enroll patients with anemia, ischemic heart disease, or active bleeding. Walsh and colleagues [39] enrolled 100 ventilated ICU patients in a prospective, randomized study and found a non-significant trend toward lower mortality in the restrictive group (Hb trigger 70 g/L, target 71 to 90 g/L) compared with the liberal group (Hb trigger 90 g/L, target 91 to 110 g/L) 180 days after randomization (RR 0.68, 95% CI 0.44 to 1.05; *P* = 0.073). Thirty-two percent of the patients had ischemic heart disease. Surely, more studies will follow and hopefully provide a more definite answer as to what is safe for patients with cardiovascular diseases. Finally, we also recommend a strict ‘single unit policy’ with re-evaluation of the clinical situation after each unit transfused except in cases of massive bleeding.

## Barriers to implementation

Synthesizing and implementing new standards of care can be very demanding. Great importance should be placed on the education of staff to change attitudes toward blood testing. It is vital to address knowledge gaps, facilitate behaviors, and reduce barriers to them. The implementation of checklists alongside training sessions of care-providing staff is a highly recommended tool to maintain adherence to new standards. Table 1 summarizes the different measures to limit blood loss in critically ill patients. The desired laboratory ordering behavior and the employment of blood-sparing techniques should be reinforced.

**Table 1:** Measures to spare patient blood

**Phlebotomy losses**	**Coagulation management**	**Further approaches**
• Quantify sampling volume and frequency	• Maintain optimal hemostatic conditions (pH, Ca^2+^, and temperature)	• Non-invasive monitoring
• Critically assess parameters ordered	• Algorithm-based hemotherapy	• Citrate-anticoagulated dialysis
• Microchemistry techniques, possibly pediatric vials		• Cell salvage
• Point-of-care bedside tests		• Education and checklists
• In-line blood sampling devices		

However, one should be aware of barriers to implementation. Further investigation of routine laboratory orderings may lead to the conclusion that many orderings are due primarily to real or perceived medico-legal reasons. Sincere and powerful fear of medical malpractice claims may lead to superfluous testing and controlling of more parameters than medically indicated, in order to minimize legal risks. Therefore, we would recommend engagement in introspective and collegial dialogue over professional practice and litigation risk factors to strive for better patient outcomes while reducing burdens on the health-care system. We suggest that inconsistencies be questioned, diagnostic ordering routines be revised, and anxieties concerning exposure to potential litigation and legal liability be minimized, for the benefit of our patients’ clinical outcome. Furthermore, economic arguments seem to increasingly permeate many aspects of hospital routine. Although these should not guide our medical decision-making, they are vital to consider as resources are finite. Personnel and material costs need to be weighed against potential savings from better patient outcome. Limiting the use of laboratory order sets will require more attention from the whole team involved in ICU therapy, in addition to thoughtful guidance by senior physicians. It is true that individualized laboratory ordering may be more resource-intensive and also may require manual processing by the laboratory staff, especially with the use of smaller pediatric vials. In our opinion, savoring every drop of blood even has the potential to reduce costs. This is applicable both directly to blood transfusion units and to the wider economic impact of associated adverse events, from both hospital-acquired anemia and allogeneic blood transfusions [40,41]: delayed wound healing, nosocomial infections, renal insufficiency, and major adverse cardiac events, to name a few. Therefore, a medico-economic evaluation of the effects of the implementation of a bundle of measurements is worthwhile and indeed may favor the improved standards of care described here.

In conclusion, blood use in ICUs can be excessive for various reasons. Developing and adopting better standards of care can ameliorate the vicious cycle we find ourselves in now, in which superfluous testing can result in iatrogenic anemia that may necessitate subsequent RBC transfusion. The rate of allogeneic blood transfusions can be further minimized by employing restrictive transfusion policies. Combined, these multimodal patient blood management concepts have great potential to avoid transfusion-related adverse events.

Let us strive to reduce the need for allogeneic blood transfusions by preserving patients’ blood and transfuse only when absolutely necessary in order improve the quality and economy of critical care. Let us savor every drop just as a mosquito would and not drain blood from our patients like Dracula!

## Abbreviations

ABG: Arterial blood gas; CRP: C-reactive protein; Hb: Hemoglobin; POC: Point-of-care; RBC: Red blood cell; RR: Relative risk.

## Competing interests

The authors implemented the measures explained in the article in the framework of a Patient Blood Management Program at the University Hospital Frankfurt. This work is supported by departmental funding and by Vifor Pharma Deutschland GmbH (Muenchen, Germany), B. Braun Melsungen AG (Melsungen, Germany), CSL Behring (Marburg, Germany), and Fresenius Kabi (Bad Homburg, Germany). The authors declare that they have no further competing interests.
